# Use of Diabetes Technologies and Retinopathy in Adults With Type 1 Diabetes

**DOI:** 10.1001/jamanetworkopen.2024.0728

**Published:** 2024-03-06

**Authors:** T. Y. Alvin Liu, Julia Shpigel, Fatima Khan, Kerry Smith, Laura Prichett, Roomasa Channa, Sarah Kanbour, Marissa Jones, Mohammed S. Abusamaan, Aniket Sidhaye, Nestoras Mathioudakis, Risa M. Wolf

**Affiliations:** 1 Wilmer Eye Institute, Johns Hopkins University School of Medicine, Baltimore, Maryland; 2 Division of Pediatric Endocrinology, Johns Hopkins University School of Medicine, Baltimore, Maryland; 3 Epidemiology and Data Management (BEAD) Core, Johns Hopkins School of Medicine Biostatistics, Baltimore, Maryland; 4 Department of Ophthalmology, University of Wisconsin, Madison; 5 Division of Endocrinology, Diabetes, & Metabolism, Johns Hopkins University School of Medicine, Baltimore, Maryland

## Abstract

**Importance:**

Diabetic retinopathy (DR) is a complication of diabetes that can lead to vision loss. Outcomes of continuous glucose monitoring (CGM) and insulin pump use in DR are not well understood.

**Objective:**

To assess the use of CGM, insulin pump, or both, and DR and proliferative diabetic retinopathy (PDR) in adults with type 1 diabetes (T1D).

**Design, Setting, and Participants:**

A retrospective cohort study of adults with T1D in a tertiary diabetes center and ophthalmology center was conducted from 2013 to 2021, with data analysis performed from June 2022 to April 2023.

**Exposure:**

Use of diabetes technologies, including insulin pump, CGM, and both CGM and insulin pump.

**Main Outcomes and Measures:**

The primary outcome was development of DR or PDR. A secondary outcome was the progression of DR for patients in the longitudinal cohort. Multivariable logistic regression models assessed for development of DR and PDR and association with CGM and insulin pump use.

**Results:**

A total of 550 adults with T1D were included (median age, 40 [IQR, 28-54] years; 54.4% female; 24.5% Black or African American; and 68.4% White), with a median duration of diabetes of 20 (IQR, 10-30) years, and median hemoglobin A_1c_ (HbA_1c_) of 7.8% (IQR, 7.0%-8.9%). Overall, 62.7% patients used CGM, 58.2% used an insulin pump, and 47.5% used both; 44% (244 of 550) of the participants had DR at any point during the study. On univariate analysis, CGM use was associated with lower odds of DR and PDR, and CGM with pump was associated with lower odds of PDR (all *P* < .05), compared with no CGM use. Multivariable logistic regression adjusting for age, sex, race and ethnicity, diabetes duration, microvascular and macrovascular complications, insurance type, and mean HbA_1c_, showed that CGM was associated with lower odds of DR (odds ratio [OR], 0.52; 95% CI, 0.32-0.84; *P* = .008) and PDR (OR, 0.42; 95% CI, 0.23-0.75; *P* = .004), compared with no CGM use. In the longitudinal analysis of participants without baseline PDR, 79 of 363 patients (21.8%) had progression of DR during the study.

**Conclusions and Relevance:**

In this cohort study of adults with T1D, CGM use was associated with lower odds of developing DR and PDR, even after adjusting for HbA_1c_. These findings suggest that CGM may be useful for diabetes management to mitigate risk for DR and PDR.

## Key Points

**Question:**

Are continuous glucose monitoring (CGM) and insulin pump use associated with the development of diabetic retinopathy (DR)?

**Findings:**

In this cohort study including 550 individuals with type 1 diabetes, CGM use was associated with lower odds of developing DR and proliferative DR. During the 8-year study period, in the age of diabetes technology, 21.8% of adults with type 1 diabetes in this cohort experienced progression of DR.

**Meaning:**

The findings of this study suggest that CGM should be encouraged for diabetes management, as it is associated with lower odds of developing DR.

## Teaser

This cohort study examines the association of use of continuous glucose monitoring and insulin pumps with diabetic retinopathy among patients with type 1 diabetes.

## Introduction

Diabetic retinopathy (DR), a debilitating complication of diabetes, is the leading cause of irreversible blindness in the working-age population in the world.^1,2,3,4^ It is well established that higher hemoglobin A_1c_ (HbA_1c_) and longer duration of diabetes are major risk factors for DR development and progression in patients with both type 1 diabetes (T1D) and type 2 diabetes (T2D).^5^ Compared with adults with T2D, adults with T1D are particularly at risk for DR complications, due to earlier onset and inherent glycemic variability associated with T1D.^5^ The landmark Diabetes Control and Complications Trial offered meaningful insights into the progression and prevention of DR. It revealed that without intensive insulin therapy, an alarming proportion—nearly 50%—of patients with T1D developed DR within a mean span of 6.5 years. Yet, the study also suggested that intensive insulin therapy, coupled with reduced HbA_1c_ levels, could significantly decrease both the risk and progression of DR.^5^

In the past 2 decades, we have witnessed the emergence and proliferation of diabetes technologies such as continuous glucose monitors (CGMs) and insulin pumps.^6^ These tools, now integral to modern diabetes management, are associated with lower HbA_1c_ levels, decreased incidence of diabetic ketoacidosis, and reduced hypoglycemic events.^7,8,9,10,11^ Prior studies have reported an association of insulin pump use with a reduced risk of DR in adolescents and individuals who are pregnant.^12,13,14,15^ Furthermore, studies in adults with T2D using CGM have shown that less time spent in the target glucose level range is associated with DR, yet there are limited data available on CGM use and association with diabetes complications in individuals with T1D.^16,17^

However, a major knowledge gap persists: data on the potential outcomes associated with diabetes-related complications in patients using CGMs, particularly among adults with T1D, are scant. To address this gap, this study aimed to examine whether the use of CGM, insulin pump, or a combination of both, is associated with a lower risk of DR, including its severe form, proliferative diabetic retinopathy (PDR), in patients with T1D. We hypothesized that the use of diabetes technologies is associated with reduced risk of DR.

## Methods

### Patients and Design

This study was a retrospective, electronic medical record (EMR)–based longitudinal cohort study of adults with T1D who had consultations at both the Johns Hopkins Endocrine and Diabetes Center and the Wilmer Eye Institute in Baltimore, Maryland, from 2013 to 2021. Inclusion criteria were a confirmed T1D diagnosis based on the *International Statistical Classification of Diseases, Tenth Revisions*, *Clinical Modification* (*ICD-10*) codes (E10.xxx) and recorded visits at both clinics. Historical *International Classification of Diseases, 9th Revision* codes were automatically converted to *ICD-10* codes in the EMR. Patients were excluded if they had other forms of diabetes (T2D, maturity-onset diabetes of youth, cystic fibrosis–related diabetes), absence of insulin requirement following pancreas transplant for T1D, or absence of HbA_1c_ data during the study period. The study received approval by the Johns Hopkins University School of Medicine Institutional Review Board according to the Declaration of Helsinki for retrospective data analysis; since it would not be practical or feasible to obtain consents for retrospective medical record reviews, a waiver of consent was granted. The Strengthening the Reporting of Observational Studies in Epidemiology (STROBE) reporting guideline was followed.

In the first analysis to examine the association of diabetes technology use with DR, patients who had visits in both the diabetes center and the ophthalmology clinics and were using CGM at the start of the study period or started CGM during the active study period (before the last ophthalmology encounter or before a diagnosis of DR) were included. Patients who started CGM after their last ophthalmology encounter or after they received a diagnosis of DR were excluded.

The second analysis was longitudinal, assessing progression of DR, and included patients who had at least 2 consecutive visits in the ophthalmology clinic and did not have proliferative DR at the initial ophthalmology visit. Patients with no DR at baseline or non-PDR at baseline were analyzed for DR progression (Figure).

**Figure.**
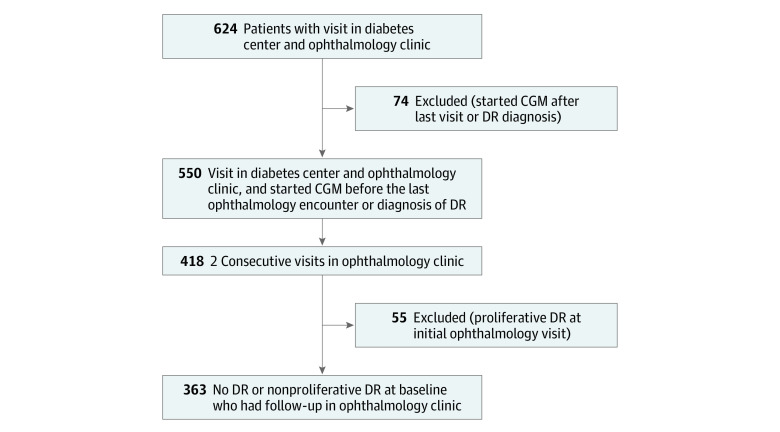
Flowchart of Patient Inclusion and Exclusion in Analyses CGM indicates continuous glucose monitoring; DR, diabetic retinopathy.

### Diabetes and Clinical Characteristics

Patient data were systematically gathered using an automated extraction from the EMR (EpicCare) supplemented with manual EMR reviews. This information covered demographic and clinical aspects, such as age; gender; race and ethnicity; socioeconomic factors, including Area Deprivation Index (ADI); diabetes technology use; microvascular complications (other than DR); macrovascular complications; and health outcomes.^18^ Race and ethnicity data were captured from the EMR and based on patient self-report, and categorized as Black or African American, White, and Other. Other was defined as those who identified as Alaska Native, Asian, Native American, Native Hawaiian, Other Pacific Islander, or Other. Unknown was defined as those who identified as unknown or declined to answer. Ethnicity was categorized as not Hispanic or Latino, or Hispanic, Latinx, or Other. The 2018 version of the ADI, consisting of 17 measures of employment, housing quality, and poverty originally extracted from long-form US census data, was used to measure patients’ neighborhood socioeconomic status via linked zip codes.

Since we excluded patients who started CGM after the study period or after they had received a diagnosis of DR, all patients designated as using CGM in this study either entered the study period using CGM or started using CGM during the study period. If the participant started CGM use during the study period, a CGM start date was verified in the EMR.

### Ophthalmology Data

We collected ophthalmology data from patients diagnosed with T1D who visited the Wilmer Eye Institute ophthalmology clinic. Diagnosis codes and diagnosis, including the presence or absence of DR or diabetic macular edema, were extracted from the EMR and then manually reviewed for accuracy by 2 independent reviewers (J.S. and F.K.), with discrepancies adjudicated by a board-certified retinal specialist (T.Y.A.L.). The diagnosis of DR was determined based on the highest level of DR in the worse eye. The presence and severity of DR were determined using specific key words (microaneurysm; intraretinal hemorrhage/heme; cotton wool spots; lipid; venous beading; macular edema, fluid, or thickening; preretinal hemorrhage; vitreous hemorrhage; neovascularization; fibrovascular proliferation, tractional retinal detachment, panretinal photocoagulation, and laser). Diabetic retinopathy was further categorized into 5 groups: none, nonproliferative without macular edema, nonproliferative with macular edema, proliferative without macular edema, and proliferative with macular edema. If DR was recorded at the first eye visit, but not reported at the follow-up visit, the retinal specialist reviewed the record to determine the presence of DR. For encounters without a retina examination, the retinal specialist manually extracted data from the nearest ophthalmology encounter and retina examination.

### Outcomes

The primary outcome of interest was the development of DR or PDR. A secondary outcome was the progression of DR for patients with more than 1 ophthalmology encounter in the longitudinal dataset. Progression was defined as the development of DR after no diagnosis of DR at the initial encounter or development of PDR after a diagnosis of non-PDR at the initial encounter. The exposure of interest was the use of diabetes technologies, including insulin pump use, CGM use, and both CGM and insulin pump use.

### Statistical Analysis

Data analysis was performed from June 2022 to April 2023. Demographic and clinical characteristics were compared between patients with and without exposure to diabetes technologies (including CGM alone, insulin pump alone, and combined CGM and pump), using Wilcoxon rank sum tests for continuous variables and Pearson χ^2^ tests for categorical data. Kruskal-Wallis tests and Pearson χ^2^ tests were used to compare demographic and clinical characteristics of patients with no DR, non-PDR without macular edema, non-PDR with macular edema, PDR without macular edema, and PDR with macular edema.

The outcomes of interest (DR and PDR) and demographic and clinical characteristics were also evaluated using a series of univariate and multivariable logistic regression models. Multivariable logistic regression models for the outcomes of DR and PDR and inclusion and exclusion of variables in the model were based on published studies, clinical relevance, and statistical considerations (characteristics with an association [*P* < .05]) with each outcome in the univariate analysis). Multivariable logistic regression included demographic variables (age, race and ethnicity, and insurance type as a proxy for socioeconomic status), clinical factors (mean HbA_1c_ level and presence of other microvascular and macrovascular complications), and other relevant variables that could be associated with the exposure and outcome (duration of diabetes). Demographic variables of marital status, ADI rank, and employment status were not included due to their collinearity with insurance type. Univariate regression analysis and multivariable logistic regression were similarly conducted for the outcome of progression of DR. We applied inverse probability–weighted regression adjustment, using the identical set of variables as used in the multivariate logistic regression analysis, to address potential confounding from who chose to use CGM or bias in those selected to receive CGM. To account for the increased risk of type 1 errors associated with conducting multiple statistical tests in the univariate analysis, we implemented the Bonferroni correction to adjust the significance threshold to *P* < .003 with 2-sided, unpaired analysis, providing a more stringent criterion for determining statistical significance. Statistical analysis was performed with Stata, version 17 (StataCorp LLC).

## Results

### Clinical and Demographic Characteristics

A total of 624 adults with T1D were identified as having a visit in both the diabetes clinic and the ophthalmology clinic. Of these, 550 patients entered the study period using a CGM or started using one during the study period and before they received a diagnosis of DR. As reported in Table 1 and the eTable in Supplement 1, the median age of the total cohort was 40 (IQR, 28-54) years, 299 (54.4%) were female and 251 (45.6%) were male, with a median duration of diabetes of 20 (IQR, 10-30) years, and a median HbA_1c_ of 7.8% (IQR, 7.0%-8.9%). During the study period, 345 patients (62.7%) used CGM, 320 (58.2%) used an insulin pump, and 261 (47.5%) used both a CGM and insulin pump. A total of 135 patients (24.5%) were Black/African American, 27 (4.9%) were Hispanic, and 376 (68.4%) were White. Most patients (396 [72.0%]) had private or commercial insurance and 301 (54.7%) were employed. Patients were equally distributed across the ADI scores by quintile.

**Table 1.** Patient Characteristics by Exposure Category for Diabetes Technologies^a^

Factor	Total, No. (%) (N = 550)	CGM, No. (%)		*P* value	Pump, No. (%)		*P* value	CGM plus pump, No. (%)		*P* value
		No (n = 205)	Yes (n = 345)		No (n = 230)	Yes (n = 320)		No (n = 289)	Yes (n = 261)	
Age at first encounter-median (IQR)	40.0 (28.0-54.0)	41.0 (28.0-56.0)	40.0 (27.0-53.0)	.29	41.0 (30.0-55.0)	39.0 (27.0-54.0)	.17	41.0 (28.0-56.0)	38.0 (27.0-52.0)	.08
Age category, y										
1-20	40 (7.3)	15 (7.3)	25 (7.2)	.30	17 (7.4)	23 (7.2)	.91	21 (7.3)	19 (7.3)	.22
21-40	239 (43.5)	87 (42.4)	152 (44.1)		96 (41.7)	143 (44.7)		120 (41.5)	119 (45.6)	
41-60	199 (36.2)	69 (33.7)	130 (37.7)		85 (37.0)	114 (35.6)		102 (35.3)	97 (37.2)	
61-80	72 (13.1)	34 (16.6)	38 (11.0)		32 (13.9)	40 (12.5)		46 (15.9)	26 (10.0)	
Sex										
Female	299 (54.4)	109 (53.2)	190 (55.1)	.67	122 (53.0)	177 (55.3)	.60	150 (51.9)	149 (57.1)	.22
Male	251 (45.6)	96 (46.8)	155 (44.9)		108 (47.0)	143 (44.7)		139 (48.1)	112 (42.9)	
Race										
Black or African American	135 (24.5)	80 (39.0)	55 (15.9)	<.001	97 (42.2)	38 (11.9)	<.001	110 (38.1)	25 (9.6)	<.001
White	376 (68.4)	111 (54.1)	265 (76.8)		117 (50.9)	259 (80.9)		161 (55.7)	215 (82.4)	
Other^b^	35 (6.4)	14 (6.8)	21 (6.1)		16 (7.0)	19 (5.9)		18 (6.2)	17 (6.5)	
Unknown/declined to answer	4 (0.7)	0	4 (1.2)		0	4 (1.2)		0	4 (1.5)	
Ethnicity										
Not Hispanic or Latino	523 (95.1)	200 (97.6)	323 (93.6)	.04	220 (95.7)	303 (94.7)	.61	279 (96.5)	244 (93.5)	.09
Hispanic-Latinx or Other	27 (4.9)	5 (2.4)	22 (6.4)		10 (4.3)	17 (5.3)		10 (3.5)	17 (6.5)	
Diabetes duration, median (IQR), y	20.0 (10.0-30.0)	19.0 (10.0-31.0)	20.0 (10.0-30.0)	.96	16.5 (6.0-26.0)	21.0 (13.0-30.5)	<.001	18.0 (8.0-30.0)	21.0 (13.0-30.0)	.02
Weight, median (IQR), kg	75.3 (65.3-87.7)	73.3 (64.5-84.6)	76.2 (65.5-89.1)	.03	72.8 (63.6-84.2)	76.3 (66.2-89.6)	.003	73.7 (64.7-84.8)	76.5 (65.8-89.4)	.04
BMI, median (IQR)	25.8 (23.3-29.7)	25.5 (22.7-29.4)	25.8 (23.5-30.1)	.15	25.5 (22.8-29.1)	25.8 (23.5-30.3)	.08	25.6 (23.2-29.4)	25.8 (23.4-30.2)	.21
Marital status										
Married	264 (48.0)	78 (38.0)	186 (53.9)	.002	100 (43.5)	164 (51.2)	.22	120 (41.5)	144 (55.2)	.01
Single	229 (41.6)	100 (48.8)	129 (37.4)		107 (46.5)	122 (38.1)		134 (46.4)	95 (36.4)	
Divorced/separated/widowed	47 (8.5)	24 (11.7)	23 (6.7)		18 (7.8)	29 (9.1)		30 (10.4)	17 (6.5)	
Unknown/other	10 (1.8)	3 (1.5)	7 (2.0)		5 (2.2)	5 (1.6)		5 (1.7)	5 (1.9)	
ADI state rank (quintiles)										
First (1-2): least disadvantaged	109 (19.8)	24 (11.7)	85 (24.6)	<.001	36 (15.7)	73 (22.8)	<.001	44 (15.2)	65 (24.9)	<.001
Second (3-4)	82 (14.9)	24 (11.7)	58 (16.8)		30 (13.0)	52 (16.2)		37 (12.8)	45 (17.2)	
Third (5-6)	92 (16.7)	41 (20.0)	51 (14.8)		39 (17.0)	53 (16.6)		54 (18.7)	38 (14.6)	
Fourth (7-8)	92 (16.7)	39 (19.0)	53 (15.4)		38 (16.5)	54 (16.9)		51 (17.6)	41 (15.7)	
Fifth (9-10)	95 (17.3)	55 (26.8)	40 (11.6)		63 (27.4)	32 (10.0)		70 (24.2)	25 (9.6)	
Missing	80 (14.5)	22 (10.7)	58 (16.8)		24 (10.4)	56 (17.5)		33 (11.4)	47 (18.0)	
Employment Status										
Employed	301 (54.7)	92 (44.9)	209 (60.6)	<.001	106 (46.1)	195 (60.9)	<.001	140 (48.4)	161 (61.7)	<.001
Not employed	98 (17.8)	54 (26.3)	44 (12.8)		58 (25.2)	40 (12.5)		68 (23.5)	30 (11.5)	
Student	32 (5.8)	7 (3.4)	25 (7.2)		10 (4.3)	22 (6.9)		12 (4.2)	20 (7.7)	
Disabled	44 (8.0)	24 (11.7)	20 (5.8)		26 (11.3)	18 (5.6)		31 (10.7)	13 (5.0)	
Retired	60 (10.9)	23 (11.2)	37 (10.7)		24 (10.4)	36 (11.2)		32 (11.1)	28 (10.7)	
Unknown	15 (2.7)	5 (2.4)	10 (2.9)		6 (2.6)	9 (2.8)		6 (2.1)	9 (3.4)	
Insurance type										
Private	396 (72.0)	119 (58.0)	277 (80.3)	<.001	143 (62.2)	253 (79.1)	<.001	179 (61.9)	217 (83.1)	<.001
Medicare	106 (19.3)	53 (25.9)	53 (15.4)		49 (21.3)	57 (17.8)		68 (23.5)	38 (14.6)	
Medicaid	34 (6.2)	24 (11.7)	10 (2.9)		28 (12.2)	6 (1.9)		31 (10.7)	3 (1.1)	
Other	14 (2.5)	9 (4.4)	5 (1.4)		10 (4.3)	4 (1.2)		11 (3.8)	3 (1.1)	
Smoking status (smoker vs nonsmoker)	177 (32.3)	82 (40.0)	95 (27.7)	.003	86 (37.4)	91 (28.6)	.03	109 (37.7)	68 (26.3)	.004
Mean HbA_1c_, median (IQR), %^c^	7.8 (7.0-8.9)	8.4 (7.5-10.0)	7.5 (6.8-8.3)	<.001	8.4 (7.4-9.8)	7.4 (6.9-8.2)	<.001	8.3 (7.4-9.6)	7.3 (6.7-8.2)	<.001
Macrovascular complications	67 (12.2)	35 (17.1)	32 (9.3)	.01	31 (13.5)	36 (11.2)	.43	43 (14.9)	24 (9.2)	.04
Microvascular complications^d^	181 (32.9)	87 (42.4)	94 (27.2)	<.001	86 (37.4)	95 (29.7)	.06	110 (38.1)	71 (27.2)	.01
DR diagnosis	244 (44.4)	111 (54.1)	133 (38.6)	<.001	98 (42.6)	146 (45.6)	.48	134 (46.4)	110 (42.1)	.32
Proliferative DR diagnosis	97 (17.6)	54 (26.3)	43 (12.5)	<.001	47 (20.4)	50 (15.6)	.14	61 (21.1)	36 (13.8)	.03

Abbreviations: ADI, area deprivation index; BMI, body mass index (calculated as weight in kilograms divided by height in meters squared); CGM, continuous glucose monitor; DR, diabetic retinopathy; HbA_1C_, hemoglobin A_1c_.

^a^ Wilcoxon rank sum tests used for continuous variables, and Pearson χ^2^ tests used for categorical data.

^b^ Other was defined as those who identified as Alaska Native, Asian, Native American, Native Hawaiian, Other Pacific Islander, or Other.

^c^ Mean Hb_A1c_ is reported as the mean of all Hb_A1c_ values available during the study period, reported as a median (IQR) because levels are not normally distributed.

^d^ Microvascular complications do not include DR.

### Diabetic Retinopathy

As reported in Table 1, 44.4% of the patients (244 of 550) had a diagnosis of DR. Of those with DR, 49.2% (120 of 244) had non-PDR without macular edema, 11.1% (27 of 244) had non-PDR with macular edema, 27.9% (68 of 244) had PDR without macular edema, and 11.9% (29 of 244) had PDR with macular edema.

### Clinical Characteristics Associated With DR

Patient characteristics by level of severity of DR are reported in Table 2. On univariate analysis, older age (odds ratio [OR], 1.02; 95% CI, 1.01-1.03; *P* < .001), longer diabetes duration (OR, 1.06; 95% CI, 1.05-1.08; *P* < .001), higher mean HbA_1c_ (OR, 1.15; 95% CI, 1.04-1.27; *P* = .009), having Medicare insurance (OR, 2.13; 95% CI, 1.38-3.29; *P* = .001), and the presence of other microvascular (OR, 3.61; 95% CI, 2.49-5.25; *P* < .001) and macrovascular (OR, 2.71; 95% CI, 1.58-4.63; *P* < .001) complications were associated with DR, and CGM use was associated with lower odds of DR (OR, 0.53; 95% CI, 0.37-0.75; *P* < .001), compared with no CGM use. For PDR, on univariate analysis, older age (OR, 1.03; 95% CI, 1.02-1.05; *P* < .001), longer diabetes duration (OR, 1.07; 95% CI, 1.05-1.09; *P* < .001), more disadvantaged ADI quintiles (ADI 9-10th quintiles: OR, 4.19; 95% CI, 1.85-9.48; *P* = .001), and the presence of other microvascular (OR, 8.52; 95% CI, 5.18-14.01; *P* < .001) and macrovascular (OR, 3.68; 95% CI, 2.12-6.39; *P* < .001) complications were associated with PDR, and Medicare (OR, 4.71; 95% CI, 2.83-7.84; *P* < .001) and Medicaid (OR, 3.03; 95% CI, 1.33-6.93; *P* = .008) insurance were associated with PDR in comparison with private or commercial insurance.

**Table 2.** Patient Characteristics by Level of Severity of Diabetic Retinopathy (DR)^a^

Factor	Total, No. (%) (N = 550)	No DR, No. (%) (n = 306)	Nonproliferative DR, No. (%)		Proliferative DR, No. (%)		*P* value
			Without ME (n = 120)	With ME (n = 27)	Without ME (n = 68)	With ME (n = 29)	
Age at first encounter, median (IQR), y	40.0 (28.0-54.0)	37.0 (26.0-51.0)	40.5 (27.0-55.5)	46.0 (33.0-58.0)	51.5 (38.0-58.0)	48.0 (30.0-59.0)	<.001
Age category, y							
1-20	40 (7.3)	30 (9.8)	9 (7.5)	0	1 (1.5)	0	.01
21-40	239 (43.5)	142 (46.4)	51 (42.5)	12 (44.4)	20 (29.4)	14 (48.3)	
41-60	199 (36.2)	104 (34.0)	40 (33.3)	11 (40.7)	35 (51.5)	9 (31.0)	
61-80	72 (13.1)	30 (9.8)	20 (16.7)	4 (14.8)	12 (17.6)	6 (20.7)	
Race							
Black or African American	135 (24.5)	71 (23.2)	25 (20.8)	8 (29.6)	24 (35.3)	7 (24.1)	.28
White	376 (68.4)	213 (69.6)	88 (73.3)	18 (66.7)	40 (58.8)	17 (58.6)	
Other^b^	35 (6.4)	21 (6.9)	6 (5.0)	1 (3.7)	3 (4.4)	4 (13.8)	
Unknown/declined to answer	4 (0.7)	1 (0.3)	1 (0.8)	0	1 (1.5)	1 (3.4)	
Ethnicity							
Not Hispanic or Latino	523 (95.1)	287 (93.8)	117 (97.5)	27 (100.0)	65 (95.6)	27 (93.1)	.37
Hispanic, Latinx or other	27 (4.9)	19 (6.2)	3 (2.5)	0	3 (4.4)	2 (6.9)	
Diabetes duration, median (IQR), y	20.0 (10.0-30.0)	14.0 (6.0-23.0)	23.0 (14.5-33.0)	20.0 (12.0-30.0)	36.0 (24.5-45.5)	26.0 (21.0-35.0)	<.001
Weight, median (IQR), kg	75.3 (65.3-87.7)	75.3 (65.3-85.9)	78.1 (66.0-92.2)	69.8 (62.6-83.0)	73.1 (60.0-88.3)	75.2 (68.9-81.3)	.18
BMI, median (IQR)	25.8 (23.3-29.7)	25.4 (23.0-29.4)	26.6 (24.6-30.4)	24.8 (22.6-27.0)	25.8 (22.6-30.9)	26.8 (24.1-28.4)	.04
Marital status							
Married	264 (48.0)	148 (48.4)	63 (52.5)	13 (48.1)	27 (39.7)	13 (44.8)	.03
Single	229 (41.6)	133 (43.5)	46 (38.3)	13 (48.1)	25 (36.8)	12 (41.4)	
Divorced/separated/widowed	47 (8.5)	22 (7.2)	8 (6.7)	1 (3.7)	14 (20.6)	2 (6.9)	
Unknown/other	10 (1.8)	3 (1.0)	3 (2.5)	0	2 (2.9)	2 (6.9)	
Area deprivation index state rank (quintiles)							
First (1-2): least disadvantaged	109 (19.8)	67 (21.9)	30 (25.0)	3 (11.1)	6 (8.8)	3 (10.3)	.29
Second (3-4)	82 (14.9)	44 (14.4)	17 (14.2)	4 (14.8)	12 (17.6)	5 (17.2)	
Third (5-6)	92 (16.7)	52 (17.0)	21 (17.5)	3 (11.1)	13 (19.1)	3 (10.3)	
Fourth (7-8)	92 (16.7)	47 (15.4)	23 (19.2)	6 (22.2)	10 (14.7)	6 (20.7)	
Fifth (9-10)	95 (17.3)	49 (16.0)	13 (10.8)	7 (25.9)	19 (27.9)	7 (24.1)	
Missing	80 (14.5)	47 (15.4)	16 (13.3)	4 (14.8)	8 (11.8)	5 (17.2)	
Employment status							
Employed	301 (54.7)	173 (56.5)	75 (62.5)	14 (51.9)	29 (42.6)	10 (34.5)	<.001
Not employed	98 (17.8)	48 (15.7)	21 (17.5)	6 (22.2)	15 (22.1)	8 (27.6)	
Student	32 (5.8)	27 (8.8)	5 (4.2)	0	0	0	
Disabled	44 (8.0)	24 (7.8)	3 (2.5)	2 (7.4)	13 (19.1)	2 (6.9)	
Retired	60 (10.9)	27 (8.8)	14 (11.7)	3 (11.1)	10 (14.7)	6 (20.7)	
Unknown	15 (2.7)	7 (2.3)	2 (1.7)	2 (7.4)	1 (1.5)	3 (10.3)	
Insurance type							
Private or commercial	396 (72.0)	242 (79.1)	91 (75.8)	21 (77.8)	28 (41.2)	14 (48.3)	<.001
Medicare	106 (19.3)	45 (14.7)	19 (15.8)	4 (14.8)	30 (44.1)	8 (27.6)	
Medicaid	34 (6.2)	15 (4.9)	8 (6.7)	2 (7.4)	5 (7.4)	4 (13.8)	
Other	14 (2.5)	4 (1.3)	2 (1.7)	0	5 (7.4)	3 (10.3)	
Smoking status (smoker vs nonsmoker)	177 (32.3)	93 (30.5)	38 (31.7)	10 (37.0)	23 (34.3)	13 (44.8)	.56
Mean HbA_1c_, median (IQR), %^c^	7.8 (7.0-8.9)	7.7 (6.9-8.7)	7.7 (7.1-8.9)	8.7 (7.7-9.8)	8.1 (7.0-9.6)	8.2 (7.3-9.2)	.02
Macrovascular complications	67 (12.2)	23 (7.5)	10 (8.3)	8 (29.6)	21 (30.9)	5 (17.2)	<.001
Microvascular complications^d^	181 (32.9)	63 (20.6)	33 (27.5)	14 (51.9)	52 (76.5)	19 (65.5)	<.001
continuous glucose monitor use	345 (62.7)	212 (69.3)	78 (65.0)	12 (44.4)	31 (45.6)	12 (41.4)	<.001
Insulin pump use	320 (58.2)	174 (56.9)	81 (67.5)	15 (55.6)	36 (52.9)	14 (48.3)	.16
Continuous glucose monitor plus pump use	261 (47.5)	151 (49.3)	64 (53.3)	10 (37.0)	26 (38.2)	10 (34.5)	.11

Abbreviations: BMI, body mass index (calculated as weight in kilograms divided by height in meters squared); HbA_1c_, hemoglobin A_1c_; ME, macular edema.

^a^ Wilcoxon rank sum tests used for continuous variables, and Pearson χ^2^ tests for categorical data.

^b^ Other was defined as those who identified as Alaska Native, Asian, Native American, Native Hawaiian, Other Pacific Islander, or Other.

^c^ Mean Hb_A1c_ is reported as the mean of all Hb_A1c_ values available during the study period, reported as a median (IQR) because levels are not normally distributed.

^d^ Microvascular complications do not include DR.

### Diabetes Technology Use and Association With DR

 On univariate analysis, CGM use was associated with lower odds of DR (OR, 0.53; 95% CI, 0.37-0.75; *P* < .001), and lower odds of PDR (OR, 0.40; 95% CI, 0.26-0.62; *P* < .001), compared with no CGM use. While insulin pump use alone was not associated with DR (*P* = .48), CGM and pump use together were associated with lower odds of PDR (OR, 0.60; 95% CI, 0.38-0.94; *P* = .03), compared with no CGM or insulin pump use (Table 3).

**Table 3.** Factors Associated With Outcomes of DR and PDR on Univariate Logistic Regression Analysis in 550 Patients

Characteristic	Outcome of DR, OR (95% CI)	*P* value	Outcome of PDR, OR (95% CI)	*P* value
Age at first encounter	1.02 (1.01-1.03)	<.001	1.03 (1.02-1.05)	<.001
Age category, y				
1-20	1 [Reference]	NA	NA	NA
21-40	2.05 (0.96-4.39)	.065	6.47 (0.86-48.66)	.07
41-60	2.74 (1.27-5.91)	.010	11.07 (1.48-82.87)	.02
61-80	4.20 (1.79-9.88)	.001	13.00 (1.67-101.53)	.01
Sex				
Female	0.91 (0.65-1.27)	.563	0.85 (0.54-1.32)	.46
Male				
Race				
White	1 [Reference]	NA	NA	NA
Black or African American	1.18 (0.79-1.75)	.416	1.67 (1.02-2.72)	.04
Other	0.87 (0.43-1.77)	.702	1.40 (0.58-3.36)	.45
Unknown/declined to answer	3.92 (0.40-38.04)	.239	5.60 (0.77-40.54)	.09
Ethnicity				
Not Hispanic or Latino	1 [Reference]	NA	NA	NA
Hispanic, Latinx or other	0.51 (0.22-1.19)	.120	1.07 (0.39-2.89)	.90
Diabetes duration, y	1.06 (1.05-1.08)	<.001	1.07 (1.05-1.09)	<.001
Weight, kg	1.00 (0.99-1.01)	.813	0.99 (0.98-1.01)	.30
BMI	1.02 (0.99-1.05)	.218	1.00 (0.96-1.04)	.94
Primary language English	0.40 (0.07-2.17)	.286	0.42 (0.08-2.34)	.33
Marital status				
Married	1 [Reference]	NA	NA	NA
Single	0.92 (0.64-1.32)	.652	1.08 (0.66-1.76)	.76
Divorced/separated/widowed	1.45 (0.78-2.70)	.242	2.89 (1.45-5.77)	.003
Unknown/other	2.98 (0.75-11.77)	.120	3.73 (1.01-13.82)	.049
ADI state rank (quintiles)				
First (1-2): least disadvantaged	1 [Reference]	NA	NA	NA
Second (3-4)	1.38 (0.77-2.46)	.279	2.91 (1.22-6.91)	.02
Third (5-6)	1.23 (0.70-2.16)	.477	2.34 (0.98-5.58)	.06
Fourth (7-8)	1.53 (0.87-2.68)	.140	2.34 (0.98-5.58)	.06
Fifth (9-10)	1.50 (0.86-2.62)	.156	4.19 (1.85-9.48)	.001
Missing	1.12 (0.62-2.02)	.706	2.16 (0.87-5.33)	.10
Employment status				
Employed	1 [Reference]	NA	NA	NA
Not employed	1.41 (0.89-2.22)	.143	2.06 (1.16-3.66)	.01
Student	0.25 (0.09-0.67)	.006	1.00 (0.00-0.00)	NA
Disabled	1.13 (0.60-2.13)	.714	3.48 (1.71-7.06)	<.001
Retired	1.65 (0.95-2.89)	.078	2.44 (1.26 4.74)	.01
Unknown	1.55 (0.55-4.37)	.412	2.44 (0.74-8.05)	.14
Insurance type				
Private	1 [Reference]	NA	NA	NA
Medicare	2.13 (1.38-3.29)	.001	4.71 (2.83-7.84)	<.001
Medicaid	1.99 (0.98-4.03)	.056	3.03 (1.33-6.93)	.01
Other	3.93 (1.21-12.75)	.023	11.24 (3.72-33.96)	<.001
Smoking status (smoker vs nonsmoker)	1.20 (0.84-1.73)	.311	1.32 (0.84-2.09)	.23
Mean HbA_1c_	1.15 (1.04-1.27)	.009	1.11 (0.98-1.26)	.10
Macrovascular complications	2.71 (1.58-4.63)	<.001	3.68 (2.12-6.39)	<.001
Microvascular complications^a^	3.61 (2.49-5.25)	<.001	8.52 (5.18-14.01)	<.001
Total No. of endocrine encounters	1.02 (0.99-1.05)	.179	1.00 (0.98-1.04)	.59
CGM and pump				
No CGM or insulin pump use	1 [Reference]	NA	NA	NA
CGM use	0.53 (0.37-0.75)	<.001	0.40 (0.26-0.62)	<.001
Insulin pump use	1.13 (0.80-1.59)	.483	0.72 (0.46-1.12)	.15
CGM and pump use	0.84 (0.60-1.18)	.320	0.60 (0.38-0.94)	.03

Abbreviations: ADI, area deprivation index; BMI, body mass index; CGM, continuous glucose monitor; DR, diabetic retinopathy; HbA_1c_, hemoglobin A_1c_; NA, not applicable; OR, odds ratio; PDR, proliferative DR.

^a^ Microvascular complications do not include DR.

In a multivariable logistic regression analysis adjusting for age, sex, race and ethnicity, duration of diabetes, insurance type, microvascular and macrovascular complications, and mean HbA_1c_, CGM use was associated with lower odds for DR (OR, 0.52; 95% CI, 0.32-0.84; *P* = .008), compared with no CGM use (Table 4). Other factors that were associated with DR development after adjustment were duration of diabetes (OR, 1.07; 95% CI, 1.04-1.09; *P* < .001), other microvascular complications (OR, 8.87; 95% CI, 5.60-14.06; *P* < .001), and mean HbA_1c_ (OR, 1.29; 95% CI, 1.11-1.50; *P* = .001). Further analysis using multivariable logistic regression with the outcome of PDR showed that CGM use was associated with lower odds of PDR (0.42; 95% CI 0.23-0.75; *P* = .004), compared with no CGM use. Incorporating inverse-probability–weighted regression adjustment in our logistic regression analysis to mitigate for potential confounding related to CGM use revealed that CGM use was associated with a lower likelihood of PDR (estimated coefficient, −0.07; SE, 0.03; *z* = −2.570; *P* = .01; 95% CI, −0.136 to −0.018), but not with DR.

**Table 4.** Association of CGM and Insulin Pump Use With DR and PDR on Multivariable Logistic Regression Analysis in 550 Patients^a^

Pump use	DR as outcome^b^		PDR as outcome^b^	
	OR (95% CI)	*P* value	OR (95% CI)	*P* value
CGM use	0.52 (0.32-0.84)	.008	0.42 (0.23-0.75)	.004
Insulin pump use	0.92 (0.56-1.52)	.754	0.63 (0.34-1.17)	.14
CGM plus pump use	0.73 (0.45-1.18)	.198	0.63 (0.34-1.16)	.14

Abbreviations: CGM, continuous glucose monitor; DR, diabetic retinopathy; OR, odds ratio; PDR, proliferative DR.

^a^ Controlling for age, sex, race and ethnicity, duration of diabetes, insurance, microvascular complications, macrovascular complications, and mean hemoglobin A_1c_.

^b^ Reference category is no CGM or insulin pump use.

### Progression of DR

There were 418 adults who had at least 2 separate ophthalmology visits, at a mean (SD) of 4.42 (2.43) years apart. Of the total 418 patients, 55 had PDR at the initial encounter, and thus were excluded from the longitudinal analysis of DR progression. Of the remaining 363 participants, 79 (21.8%) had progression of DR in the study time frame (52 of 79 from no DR to non-PDR, 15 of 79 from no DR to PDR, and 12 of 79 from non-PDR to PDR), while the remaining participants remained stable over the study period. Factors including higher HbA_1c_ (OR, 1.20; 95% CI, 1.04-1.39; *P* = .01), presence of other microvascular complications (OR, 2.71; 95% CI, 1.61-4.56; *P* < .001), and being in the fifth ADI quintile (most disadvantaged) (OR, 2.64; 95% CI, 1.22-5.75; *P* = .01) were significant risk factors for progression of DR on univariate analysis. On multivariable logistic regression analysis, higher mean Hb_A1c_ (OR, 1.24; 95% CI, 1.02-1.51 *P* = .03) and presence of other microvascular complications (OR, 5.48; 95% CI, 2.95-10.16; *P* < .001) were associated with progression of DR. There were no associations of CGM use, insulin pump use, or CGM and insulin pump use together with progression of DR on univariate or multivariate analysis.

## Discussion

In this retrospective cohort study of a diverse population of adults with T1D at a tertiary academic center, we found that CGM use was associated with lower odds of developing DR and proliferative DR. To our knowledge, this is one of the first and largest reports noting that CGM use is associated with lower odds of DR in adults with T1D, even after adjusting for HbA_1c_ levels. This was observed even without an association between insulin pump use and DR. As the use of CGM in the management of T1D continues to increase, it may help mitigate the development of DR and vision loss related to diabetes, and thus should be encouraged in the management of diabetes.

From the landmark Diabetes Control and Complications Trial in patients with T1D, it is known that longer duration of diabetes and higher HbA_1c_ levels are associated with worse long-term diabetes outcomes, including ophthalmic complications.^5^ In this study, we similarly found diabetes duration and higher HbA_1c_ levels to be associated with any DR, and PDR. Higher HbA_1c_ levels were also associated with progression of DR in this cohort. The worldwide prevalence of DR and PDR is higher in patients with T1D, compared with patients with T2D.^19^ Hence, our finding that CGM use is associated with a lower odds of DR and PDR development in patients with T1D, a higher-risk group, is important and enhances the American Diabetes Association recommendations that CGM use be considered standard of care for glucose monitoring in individuals with T1D.^20^ In this cohort, CGM use with or without an insulin pump was associated with a lower odds of PDR development, and since most irreversible blindness associated with DR is due to PDR complications, such as tractional retinal detachment and neovascular glaucoma, these findings suggest that CGM use could potentially impact visual outcomes and mitigate permanent blindness from DR.

In this analysis, we found that CGM use was independently associated with a lower likelihood of DR and PDR, even after adjusting for glycemic control assessed by HbA_1c_. Use of CGM likely results in reduced variability in glucose levels even while mean glucose levels remain the same, and this may confer further protection against diabetes complications, including DR. Although the retrospective nature of our study and lack of consistent collection of essential CGM parameters, such as time in range and glycemic variability,^21^ precluded the analysis of these CGM variables, it has been suggested that glycemic variability may be a risk factor for associated complications.^22^ A more recent study evaluated time in range as assessed by CGM in adults with T2D and found that lower time spent in the target range was associated with worse DR, independent of HbA_1c_ level or measure of glycemic variability.^16^ A recent meta-analysis found that glycemic variability and low time in range were associated with all microvascular and macrovascular complications of diabetes in individuals with T1D and T2D, yet highlighted the limited data available in T1D and need for more longitudinal studies.^17^

In our analysis, insulin pump use alone did not show an association with DR. These findings contrasted with previous studies, which reported that insulin pump use was associated with a lower likelihood of DR in the pediatric and pregnant populations.^14,15^ Other studies in adults with T1D observed a lower incidence and progression of DR and other microvascular complications (specifically albuminuria) in those who started insulin pump therapy and had a shorter diabetes duration.^12,13^ The median duration of diabetes in our cohort was longer, at 20 years, than those reported in other studies, potentially accounting for the conflicting data.^14,15^

Progression of DR was initially described in the Wisconsin Epidemiologic Study of DR^5,23^ among the intervention and conventional treatment groups over a 6.5-year (range, 3-9 years) time period, and later by the Wisconsin Diabetes Registry Study.^24,25^ However, there are limited data available on the progression of DR over the following decades when diabetes technologies became more frequently used.^6^ By analyzing the longitudinal clinical and ophthalmic data available for the 363 patients who had at least 2 ophthalmology visits during the study period, we were able to assess overall rates of DR progression using first and last encounters that were a mean (SD) of 4.42 (2.43) years apart. Overall, 21.8% of participants had some progression of DR during the study time frame, which is expectedly lower than progression rates seen in these earlier studies when use of intensive insulin therapy was being introduced and before use of diabetes technologies. Future longitudinal studies will need to more clearly elucidate the temporal association between early use of diabetes technologies and progression of DR.

### Strengths and Limitations

The strengths of this study include the diverse patient population with T1D, high use of CGM, and systematic approach to validation of ophthalmologic data collection with manual reviews of ophthalmic medical records to avoid diagnostic coding errors associated with imperfect clinical coding. The study also has limitations. Inherent to retrospective medical records reviews, results may be affected by unknown confounding factors. Although CGM use in T1D is now considered standard of care, in the earlier years of the study period, it is possible there was bias in who was selected to receive CGM that may affect results. Additionally, although CGM start date was available for all patients within the study period, insulin pump start dates were not available. Furthermore, CGM use for T1D management has increased substantially over the past decade, but since it can take much longer for DR to develop, the association of CGM use and DR may not be accurately reflected and may be an underestimate. This study period was also before the use of advanced hybrid closed-loop pump systems, which may further impact the risk for development of DR and was not able to be measured in our study. In addition, most of the patients in this cohort had private insurance and were of White race, which may not be generalizable to all adult populations with T1D.

## Conclusions

In this cohort study, CGM use in adults with T1D was associated with a reduced odds of prevalent DR and PDR. Although CGM use is considered the standard of care for glucose monitoring in T1D and its use has been increasing over the past decade, the benefits conferred by mitigating risk for diabetes-associated complications further supports the use of CGM in diabetes management. It may be useful for future research to focus on CGM-specific parameters, such as time in range and glycemic variability, as well as hybrid closed-loop insulin systems, on the reduction of complications in T1D.

## Notes

Supplement 1. **eTable.** Patient Characteristics by DR

Supplement 2.
Data Sharing Statement
