# Supplementary material for: Use of Diabetes Technologies and Retinopathy in Adults With Type 1 Diabetes
Source: JAMA Netw Open. 2024 Mar 6;7(3):e240728. doi: 10.1001/jamanetworkopen.2024.0728 (PMC10918500; doi:10.1001/jamanetworkopen.2024.0728)
Supplement: Supplement 1. — eTable. Patient Characteristics by DR [file jamanetwopen-e240728-s001.pdf]

Supplementary Online Content

Liu TYA, Shpigel J, Khan F, et al. Use of diabetes technologies and retinopathy in adults with type 1 diabetes. *JAMA Netw Open*. 2024;7(3):e240728. doi:10.1001/jamanetworkopen.2024.0728

eTable. Patient Characteristics by DR

This supplementary material has been provided by the authors to give readers additional information about their work.

© 2024 Liu TYA et al. *JAMA Network Open.*

eTable Patient Characteristics by DR Diagnosis

Factor	All Patients	No DR	Yes DR	p-value
N	550	306	244	
Age at first encounter, median (IQR)	40.0 (28.0, 54.0)	37.0 (26.0, 51.0)	44.0 (31.0, 58.0)	<0.001
Age Category (yr)				0.003
1-20	40 (7.3%)	30 (9.8%)	10 (4.1%)	
21-40	239 (43.5%)	142 (46.4%)	97 (39.8%)	
41-60	199 (36.2%)	104 (34.0%)	95 (38.9%)	
61-80	72 (13.1%)	30 (9.8%)	42 (17.2%)	
Sex				0.563
Female	299 (54.4%)	163 (53.3%)	136 (55.7%)	
Male	251 (45.6%)	143 (46.7%)	108 (44.3%)	
Race				0.48
White or Caucasian	376 (68.4%)	213 (69.6%)	163 (66.8%)	
Black or African American	135 (24.5%)	71 (23.2%)	64 (26.2%)	
Other	35 (6.4%)	21 (6.9%)	14 (5.7%)	
Unknown/Declined to Answer	4 (0.7%)	1 (0.3%)	3 (1.2%)	
Ethnicity				0.11
Not Hispanic or Latino	523 (95.1%)	287 (93.8%)	236 (96.7%)	
Hispanic, Latinx or Other	27 (4.9%)	19 (6.2%)	8 (3.3%)	
Diabetes Duration (years), median (IQR)	20.0 (10.0, 30.0)	14.0 (6.0, 23.0)	26.0 (18.0, 37.0)	<0.001
Weight (kg), median (IQR)	75.3 (65.3, 87.7)	75.3 (65.3, 85.9)	74.5 (65.3, 89.0)	0.69
BMI (kg/m2), median (IQR)	25.8 (23.3, 29.7)	25.4 (23.0, 29.4)	26.0 (23.8, 30.0)	0.049
Primary Language (English)	544 (98.9%)	304 (99.3%)	240 (98.4%)	0.27
Marital Status				0.19
Married	264 (48.0%)	148 (48.4%)	116 (47.5%)	
Single	229 (41.6%)	133 (43.5%)	96 (39.3%)	
Divorced/Separated/Widowed	47 (8.5%)	22 (7.2%)	25 (10.2%)	
Unknown/Other	10 (1.8%)	3 (1.0%)	7 (2.9%)	
ADI state rank (quintiles)				0.64
First (1-2): least disadvantaged	109 (19.8%)	67 (21.9%)	42 (17.2%)	
Second (3-4)	82 (14.9%)	44 (14.4%)	38 (15.6%)	
Third (5-6)	92 (16.7%)	52 (17.0%)	40 (16.4%)	
Fourth (7-8)	92 (16.7%)	47 (15.4%)	45 (18.4%)	
Fifth (9-10)	95 (17.3%)	49 (16.0%)	46 (18.9%)	
Missing	80 (14.5%)	47 (15.4%)	33 (13.5%)	
Employment Status				0.006
Employed	301 (54.7%)	173 (56.5%)	128 (52.5%)	
Not Employed	98 (17.8%)	48 (15.7%)	50 (20.5%)	
Student	32 (5.8%)	27 (8.8%)	5 (2.0%)	
Disabled	44 (8.0%)	24 (7.8%)	20 (8.2%)	
Retired	60 (10.9%)	27 (8.8%)	33 (13.5%)	

© 2024 Liu TYA et al. *JAMA Network Open.*

Unknown	15 (2.7%)	7 (2.3%)	8 (3.3%)	
Insurance Type				<0.001
Private	396 (72.0%)	242 (79.1%)	154 (63.1%)	
Medicare	106 (19.3%)	45 (14.7%)	61 (25.0%)	
Medicaid	34 (6.2%)	15 (4.9%)	19 (7.8%)	
Other	14 (2.5%)	4 (1.3%)	10 (4.1%)	
Smoking status (Smoker vs Non-Smoker)	177 (32.3%)	93 (30.5%)	84 (34.6%)	0.31
Mean HbA1c (%), median (IQR)*	7.8 (7.0, 8.9)	7.7 (6.9, 8.7)	8.1 (7.1, 9.3)	0.005
First HbA1c (%) in Study Period, median (IQR)	7.8 (6.8, 9.1)	7.6 (6.7, 9.0)	8.1 (7.1, 9.4)	0.005
Last HbA1c (%) in Study Period, median (IQR)	7.5 (6.7, 8.7)	7.4 (6.6, 8.5)	7.7 (6.8, 9.0)	0.024
Macrovascular Complications	67 (12.2%)	23 (7.5%)	44 (18.0%)	<0.001
Microvascular complications (No DR)**	181 (32.9%)	63 (20.6%)	118 (48.4%)	<0.001
Total Number of Diabetes Encounters, median (IQR)	7.0 (4.0, 12.0)	7.0 (3.0, 11.0)	8.0 (4.0, 13.0)	0.32
Continuous glucose monitor (CGM) use	345 (62.7%)	212 (69.3%)	133 (54.5%)	<0.001
Insulin pump use	320 (58.2%)	174 (56.9%)	146 (59.8%)	0.48
CGM and Pump use	261 (47.5%)	151 (49.3%)	110 (45.1%)	0.32

Wilcoxon rank-sum tests used for continuous variables, and Pearson's chi-squared tests for categorical data. ADI, area deprivation index. *Mean HbA1c is reported as the mean of all HbA1c values available during the study period, reported as a median (IQR). **Microvascular complications do not include diabetic retinopathy.

© 2024 Liu TYA et al. *JAMA Network Open.*
