# Supplementary material for: Use of Diabetes Technologies and Retinopathy in Adults With Type 1 Diabetes
Source: JAMA Netw Open. 2024 Mar 6;7(3):e240728. doi: 10.1001/jamanetworkopen.2024.0728 (PMC10918500; doi:10.1001/jamanetworkopen.2024.0728)
Supplement: Supplement 2. — Data Sharing Statement [file jamanetwopen-e240728-s002.pdf]

Data Sharing Statement

Liu. Use of Diabetes Technologies and Retinopathy in Adults With Type 1 Diabetes. *JAMA Netw Open*. Published March 06, 2024. doi:10.1001/jamanetworkopen.2024.0728

Data
Data available: Yes
Data types: Deidentified participant data
How to access data: Data from this study will be shared with bona fide researchers submitting a research proposal approved by the primary investigator. Data will be shared in a de-identified/anonymized format.
When available: With publication

Supporting Documents
Document types: None

Additional Information
Who can access the data: Data from this study will be shared with bona fide researchers submitting a research proposal approved by the primary investigator. Data will be shared in a de-identified/anonymized format.
Types of analyses: Secondary analysis
Mechanisms of data availability: Data from this study will be shared with bona fide researchers submitting a research proposal approved by the primary investigator. Data will be shared in a de-identified/anonymized format.
